# Changing player behaviour in sport during the COVID-19 pandemic: Shake on it?

**DOI:** 10.17159/2078-516X/2020/v32i1a8967

**Published:** 2020-01-01

**Authors:** J McKenna, S H Backhouse, G Phillips, B Jones

**Affiliations:** 1 Carnegie Applied Rugby Research (CARR) Centre, Carnegie School of Sport, Leeds Beckett University, Leeds, UK; 2 England Performance Unit, The Rugby Football League, Leeds, UK; 3 Hull Kingston Rovers, Hull, UK; 4 Leeds Rhinos Rugby League club, Leeds, UK; 5 Division of Exercise Science and Sports Medicine, Department of Human Biology, Faculty of Health Sciences, the University of Cape Town and the Sports Science Institute of South Africa, Cape Town, South Africa; 6 School of Science and Technology, University of New England, Armidale, NSW, Australia

**Keywords:** rugby, virus illness

## Abstract

To prevent the spread of infection during matches and training activities is a major challenge facing all sports returning from the enforced COVID-19 shutdown. During training and matches, rugby league players make contact with others which can result in SARS-CoV-2 virus transmission. While these interactions characterise the appeal of the game, a number of them can be avoided, including shaking hands and conversing after the match. This paper presents a framework underpinned by behavioural science (capability, opportunity, motivation and behaviour model, COM-B) to support stakeholders in helping players adopt new social distance norms and behaviours. This framework helps to ensure the players have the capability, opportunity, and motivation to adopt new COVID-19 risk minimising behaviours, which they will need to commit to 100%.

Following the return to professional sports, it must be noted that risks remain. Regardless of the sport, managing the transmission of SARS-CoV-2 is challenging. Unlike National Basketball Association (NBA) players, who completed their 2020 championship under quarantine in Disneyland, athletes and club officials from most sports come to training and competition from local communities with distinctive risk profiles. These athletes are required to follow government and National Governing Body (NGB) guidelines, while attempting to compete as normal.

In rugby league, despite the introduction of rule changes and risk mitigation strategies,^[1]^ COVID-19 outbreaks have occurred.^[2]^ What is the best way to go about planning the successful management of COVID-19 transmission through scheduled behaviour change?

As behaviour is key to preventing infection and improving outcomes, its management is crucial.^[3]^ With COVID-19, many behaviours and effective actions should be guided by behavioural science frameworks. One such framework, the Capability, Opportunity and Motivation-Behaviour (COM-B) model,^[4]^ recognises that behaviour (B) emerges from the interaction between an individual’s capability (C), opportunity (O), and motivation (M) for engaging in a specific behaviour. This model provides a guide for what needs adjustment for an effective behaviour change intervention. This model has been used within elite level sport to further understand the adherence to nutritional guidelines^[5]^ and help rugby league players achieve performance goals.^[6]^

A popular aphorism holds that 100% is easier than 98%. SARS-CoV-2 transmission thrives in groups where too many people live at the 98% level. This underlines the importance of designing systems and processes that reflect how players and coaches prefer to close that 2% gap. While simply accepting the 2% gap seems irrational, it is often predictable.^[7]^ For example, it may seem irrational for drivers to routinely turn down the radio to either reverse or to look for specific road signs. Yet without realising why, drivers do reduce the sound distraction as this helps to manage the momentary, easily overlooked but potentially lethal costs associated with loss of attention or attention-switching.

In rugby league, one challenge within the specific match day context is the post-match handshake or hug, which is a non-essential player-to-player interaction. ^[1]^ In this current scenario, it is likely that the personalities associated with the playing context are different to those of training. This puts a new and powerful meaning to the idea of game face, namely, it alters players’ responsiveness to what works on non-match days. Indeed, as evidence of their conscientiousness, players may have made substantial investments in refining the routines they believe work and may be resistant to change. They will need support to change because many are likely to feel that change will activate the worries of the neurotic tendencies that may accompany their game face.

Match days represent micro-moments (Opportunity) when all the benefits arising from self-regulation (Capability) and defaults (Motivation) of non-match days, can lose their influence. Emphasising the COM of match days may help to take the 2% out of the rugby league system, with examples illustrated in Fig. 1.

Focusing on post-match handshaking and hugging, new post-match routines at the final whistle will be important to embed the new practices into the sport’ new normal. Developing and practicing fun, easy and new ways to show respect for opponents and for the game itself, may enhance players’ motivation for change.

A complementary approach based on opportunity could change the defaults so that teams enter and leave the pitch individually rather than side by side. This contrasts with existing behaviour for example, at the final whistle, after a hard-fought match, the close proximity of the players may represent an automatic and unhelpful trigger for ubiquitous and habitual handshaking and hugs. To enhance motivation, new rewards and incentives may need to be considered for teams upholding the new defaults.

Of course, all this presupposes that infection rates arise within the context of player-to-player contact. Another complexity lies in how to prevent the virus from entering clubs. Herein lies part of the explanation for the NBA adopting a ‘closed camp’ approach. Their quarantine approach, beyond the 113-page rule book distributed to players before the season resumed, activated the power of physical opportunity via environmental restructuring i.e. they used default effects to minimise the risk of the 2% occurring. At the completion of the 2020 NBA championships, in quarantine no positive COVID-19 tests were recorded, while one player lost his contract for breaking quarantine rules.

For the NBA, their new default meant that all concerned accepted strict accountability, which is all about motivation. No one was allowed the luxury of debating or challenging their personal and collective accountability because this closes that all important 2% gap. For rugby league and other sports not in Disneyland, providing alternative safe practices that players and staff can enact through focussing on their capability, opportunity and motivation, is critical for supporting effective behaviour change that will limit the potential for high risk SARS-CoV-2 transmission situations.

## Figures and Tables

**Fig. 1:**
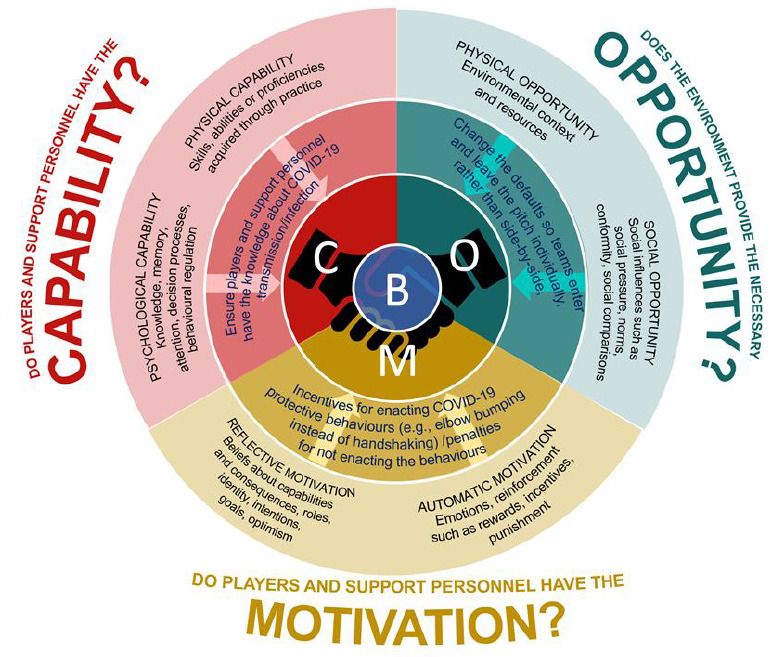
Addressing the capability, opportunity and motivation of rugby league players and support personnel to influence COVID-19 positive behaviours, adapted from Michie et al. ^[4]^
